# Spatial and taxonomic biases in bat records: Drivers and conservation implications in a megadiverse country

**DOI:** 10.1002/ece3.5848

**Published:** 2019-11-26

**Authors:** Veronica Zamora‐Gutierrez, Tatsuya Amano, Kate E. Jones

**Affiliations:** ^1^ CONACYT – Centro Interdisciplinario de Investigación para el Desarrollo Integral Regional (CIIDIR) Unidad Durango Instituto Politécnico Nacional Durango Mexico; ^2^ Conservation Science Group Department of Zoology University of Cambridge Cambridge UK; ^3^ Department of Genetics, Evolution and Environment Centre for Biodiversity and Environment Research University College London London UK; ^4^ Centre for the Study of Existential Risk University of Cambridge Cambridge UK; ^5^ School of Biological Sciences University of Queensland Brisbane Qld Australia; ^6^ Institute of Zoology Zoological Society of London London UK

**Keywords:** anthropogenic threats, chiroptera, data gaps, Mexico, risk hotspots

## Abstract

Biases in data availability have serious consequences on scientific inferences that can be derived. The potential consequences of these biases could be more detrimental in the less‐studied megadiverse regions, often characterized by high biodiversity and serious risks of human threats, as conservation and management actions could be misdirected. Here, focusing on 134 bat species in Mexico, we analyze spatial and taxonomic biases and their drivers in occurrence data; and identify priority areas for further data collection which are currently under‐sampled or at future environmental risk. We collated a comprehensive database of 26,192 presence‐only bat records in Mexico to characterize taxonomic and spatial biases and relate them to species' characteristics (range size and foraging behavior). Next, we examined variables related to accessibility, species richness and security to explain the spatial patterns in occurrence records. Finally, we compared the spatial distributions of existing data and future threats to these species to highlight those regions that are likely to experience an increased level of threats but are currently under‐surveyed. We found taxonomic biases, where species with wider geographical ranges and narrow‐space foragers (species easily captured with traditional methods), had more occurrence data. There was a significant oversampling toward tropical regions, and the presence and number of records was positively associated with areas of high topographic heterogeneity, road density, urban, and protected areas, and negatively associated with areas which were predicted to have future increases in temperature and precipitation. Sampling efforts for Mexican bats appear to have focused disproportionately on easily captured species, tropical regions, areas of high species richness and security; leading to under‐sampling in areas of high future threats. These biases could substantially influence the assessment of current status of, and future anthropogenic impacts on, this diverse species group in a tropical megadiverse country.

## Graphical abstract

We explored spatial and taxonomic information gaps on a large mammalian clade, bats; in a megadiverse country and we further identify priority areas for further data collection which are currently under‐sampled or at future environmental risk. We found that sampling efforts for Mexican bats have focused disproportionately on easily captured species, tropical regions, areas of high species richness and security; leading to under‐sampling in areas of high future threats. These biases could substantially influence the assessment of current status of, and future anthropogenic impacts on, this diverse species group in a tropical megadiverse country.

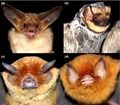

## 1 INTRODUCTION

A rapid accumulation of species occurrence data over the past several decades have enabled researchers to carry out large‐scale and multitaxa studies to develop methods for inferring present and future species distributions (Soberón & Peterson, 2004). Studies using species occurrence data provide a crucial basis for identifying areas of conservation concern, such as those with high species richness (Platts et al., 2010) and endemic or rare species (Guisan et al., 2006), evaluating the risk of invasive species and diseases (Peterson, 2003), and assessing predictors of species occurrence (Peterman, Crawford, & Kuhns, 2013). Such knowledge can then be used for identifying threats as well as prioritizing and directing future conservation efforts in this anthropozoic era (Groves et al., 2002; Peterman et al., 2013).

Ideally, species occurrence data should be uniform over space and different taxa to reflect the real variation in the distribution, richness, and abundance patterns of species. However, species occurrence data are often seriously biased toward certain regions, habitats, and environmental domains with low coverage particularly in the most biodiverse regions (Hortal, Lobo, & Jiménez‐Valverde, 2007; Soberón, Davila, & Golubov, 2004; Yesson et al., 2007). Biases in data availability have serious consequences on scientific inferences that can be derived. For example, spatial data biases can result in an uneven coverage of environmental conditions, such as biomes or climatic zones, where a species could occur (Kadmon, Farber, & Danin, 2004; Loiselle et al., 2007). Such biases can lead to deriving erroneous associations of species with environmental variables (Phillips et al., 2009; Yang, Ma, & Kreft, 2013), inferring incorrect species' absences (Bystriakova, Peregrym, Erkens, Bezsmertna, & Schneider, 2012), misidentifying areas that have been sampled intensively as species‐rich (Petřík, Pergl, & Wild, 2010) and building spurious ecological hypothesis and concepts (Hortal et al., 2015). The potential consequences of using spatially biased data could be more detrimental in less‐studied regions, often characterized by high biodiversity and serious risks of human threats, as conservation and management actions could be misdirected (Bini, Diniz‐Filho, Rangel, Bastos, & Pinto, 2006). Similarly, if threatened species are less surveyed, the uneven distribution of data over species can also lead to the underestimation of species extinction risk in the taxon (Bland, Collen, Orme, & Bielby, 2015).

Uneven sampling in environmental space is also one of the major sources of errors in studies relating species occurrence data to attributes of the environment in order to model species' niches like species distribution models (SDMs) (Kadmon, Farber, & Danin, 2003; Phillips et al., 2009). If sampling across the environmental space is uneven or if some environmental units are not sampled, the resultant model predictions will be truncated in those under‐sampled environmental conditions, which may actually be suitable (Hortal & Lobo, 2008). Model transferability is also highly sensitive to the environmental range covered by the data used to develop and parameterize models (Randin et al., 2006). Incomplete environmental response curves predicted by SDMs can be particularly problematic for environmental change studies as the full environmental tolerances of the species need to be modeled in order to accurately predict geographic changes in habitat suitability.

**Figure 1:**
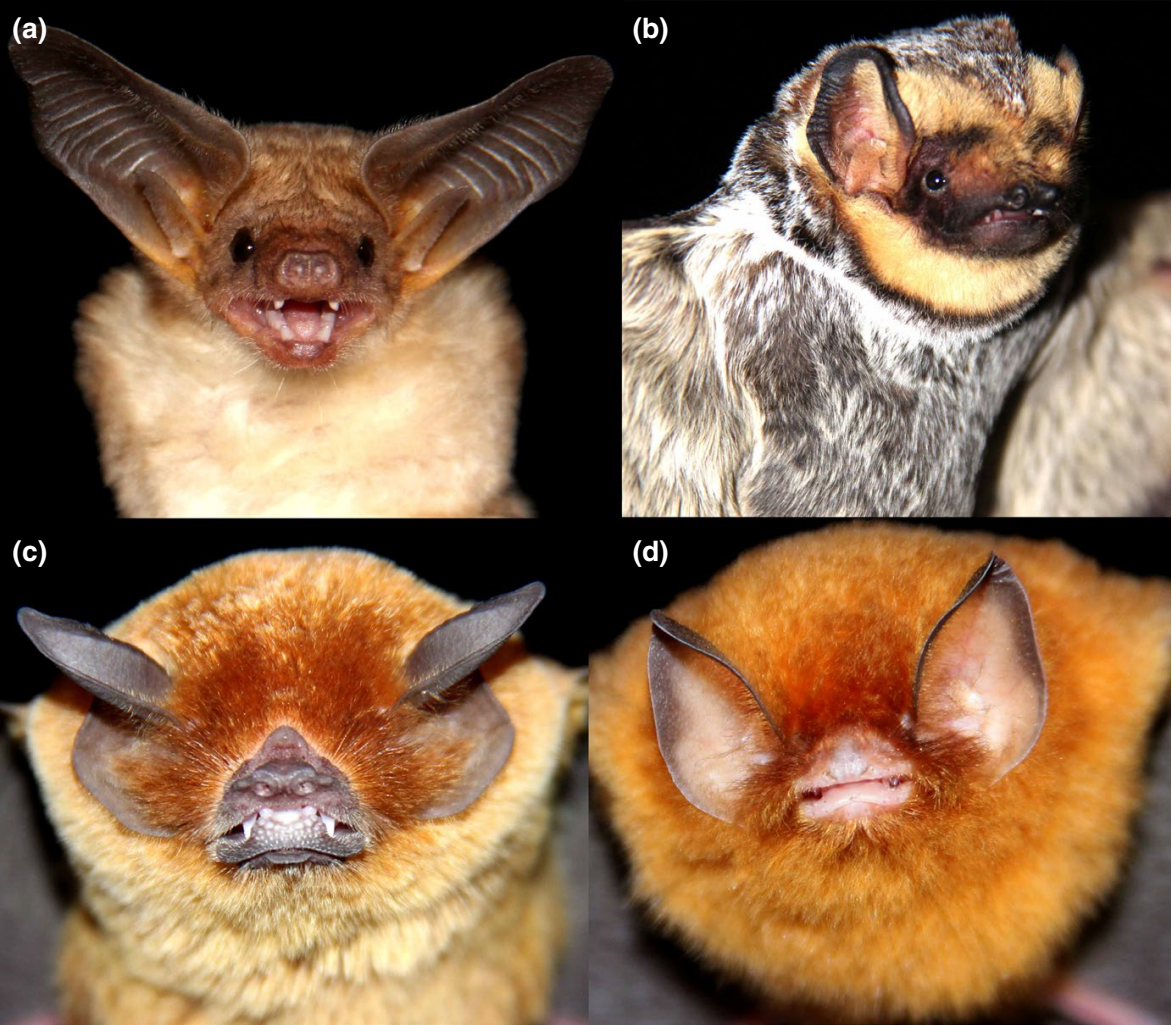
Example of the diversity of Mexican bats. (a) *Antrozous pallidus* and (b) *Lasiurus cinereus* belonging to the Vespertilionidae family, (c) *Pteronotus parnellii* from the Mormoopidae family, and (d) *Natalus mexicanus* member of the Natalidae family. Pictures by Veronica Zamora‐Gutierrez

Mexico is a megadiverse country harboring approximately 10% of all extant species (Mittermeier, Goettsch‐Mittermeier, & Robles‐Gil, 1997) and its natural capital faces serious threats caused by deforestation, species extinctions, and other environmental changes (Malcolm, Liu, Neilson, Hansen, & Hannah, 2006; Mas et al., 2004; Visconti et al., 2011). Bats include a large fraction of the richness and abundances of the Mexican mammalian faunas (~26%, Figure 1) (Ceballos & Oliva, 2005; Medellín, Arita, & Sánchez, 2008) and provide essential functions and services in ecosystems like pollination, seed dispersal, and the suppression of insects' populations (Jones, Jacobs, Kunz, Willig, & Racey, 2009). Bats are considered an ideal indicator taxon for monitoring the status, changes, and responses of biodiversity and ecosystems since their communities respond to changes in temperature and precipitation regimes, farmland expansion, and deforestation (Jones et al., 2009). However, the applicability of indicator taxa is reliant on the existence and availability of data. Although Mexico is considered to be a pioneer in biodiversity informatics (Soberón & Peterson, 2004), there are still considerable knowledge gaps and an uneven distribution of the information available (Sarukhan et al., 2015).

Here, focusing on five biases in the collection and compilation of information (taxonomic, geographical location, species richness, accessibility, and security), we aimed to explain spatial variations in the amount of available bat occurrence data in Mexico and identify priority areas where future survey efforts should be directed. We hypothesized that geographical location, species richness, accessibility, and security could have an impact in explaining spatial biases in available information and that certain groups of species will have more data available. Species occurrence data tend to be more numerous in locations that are easily accessible and less remote (e.g., near cities or with higher road and population densities), with higher actual and/or perceived biodiversity and endemism (e.g., inside protected areas, with complex landscape heterogeneity, and with reported higher species richness) and with high security levels (Amano & Sutherland, 2013; Dennis & Thomas, 2000; Kadmon et al., 2004). Similarly, there tend to be more records for species that are abundant, easily detectable (or captured) and identifiable or charismatic (Hijmans et al., 2000; Meyer et al., 2011; Newbold, 2010).

Our work provides an essential background to inform studies of macroecological patterns and reduce the risk of reaching misleading conclusions in the identification of priority conservation and risk areas. Addressing data gaps and biases is critical to assess the status of biodiversity in megadiverse countries and direct conservation efforts.

## 2 MATERIAL AND METHODS

### 2.1 Occurrence data

**Figure 2:**
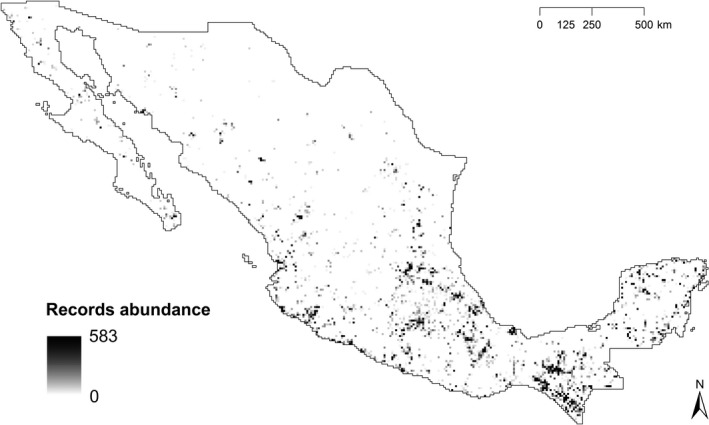
Availability of bat occurrence records. The map shows the distribution and abundance of 26,192 bat occurrence records for 134 species (98% of the known species in Mexico) and 8 families (100% of the families in Mexico) across the 2,289 5 arc minute sampled grid cells

We generated a presence‐only database for all bat species that occur in Mexico using information compiled from the National Commission for Knowledge and Use of Biodiversity (CONABIO), Global Biodiversity Information Facility (GBIF), and Mammal Networked Information System (MaNIS). We searched for published literature in English and Spanish using Web of Science, Google Scholar, and Scielo to obtain additional information on Mexican bat occurrence data using the search words “chiroptera,” “M*xico,” “bat,” “bats,” “record*,” “occurrence,” “registro*,” “quiropter*,” “murci*lago*.” Additionally, we requested unpublished material from Mexican researchers. We performed a data cleaning process as described in Zamora‐Gutierrez, Pearson, Green, and Jones (2018) leaving 26,192 occurrence records in Mexico (Figure 2) from 134 species (98% of the known species in Mexico) and eight families (100% of the families in Mexico) (Medellín et al., 2008). The total number of grid cells in Mexico was 25,833, of which 9% (2,289 cells) had at least one bat occurrence record (Figure 2). This scarcity of data translates into an area of 1.7 million km^2^ with no bat records.

### 2.2 Taxonomic bias

We assessed taxonomic sampling bias based on sampling method and species range size. Hypothetically, the number of occurrence data should increase with the size of the area in which a species occurs (Hijmans et al., 2000). To determine range size, we counted the total number of grid cells in which the species is expected to occur using species range maps (IUCN, 2015). Sampling method has been pointed before as a major factor affecting species detectability (Meyer et al., 2011). For example, phyllostomids are highly detectable by frequently used capture methods (e.g., mist nets and harp traps), while insectivorous species are mostly recorded using acoustic methods (MacSwiney, Clarke, & Racey, 2008). We used foraging space as a surrogate for sampling method because such information was not available in the datasets and assigned each species as open‐space, edge–space, or narrow‐space forager (Denzinger & Schnitzler, 2013).

We used a linear regression to determine the effects of range size (log‐transformed and centered) and foraging space (edge‐space forager as a reference category) on the number of grid cells sampled (log‐transformed) in each species, and included interaction between the two explanatory variables in the model. We then applied a Tukey HSD post hoc test to make a multiple comparison between the foraging groups, using the glht function in the multcomp package (Hothorn, Bretz, & Westfall, 2008) in R (R Development Core Team, 2013). In the same model, we estimated the slope of log‐transformed range size and its 95% confidence interval for each foraging space group using the cld function in the lsmeans package (Lenth & Love, 2016) in R. This slope can be interpreted as the exponent *b* of a power–law relationship: *y = a x^b^*, where *y* represents the number of grid cells sampled and *x* range size (i.e., the number of grid cells in which the species is expected to occur). When the estimated exponent *b* exceeds 1, it indicates that the proportions of grid cells sampled (*y*/*x* = *a x^b^* ^−1^) are high in wide‐ranging species, whereas an exponent smaller than 1 is a sign that the proportions are higher in narrow‐ranging species.

### 2.3 Spatial bias and its predictors

We first estimated the distribution of all bat occurrence records to assess whether the environmental space and the bat species in Mexico were sampled in an unbiased way. Ideally, we should also use information on abundance and species absence (i.e., a particular location was surveyed but no bat species were detected). However, neither of these types of data is usually available when collating occurrence from these repositories. We thus used the occurrence records of all bat species together as a proxy for the total bat sampling effort across Mexico. We created a grid with the extent of Mexico at a 5 arc minutes resolution (~10 km^2^) and summed the number of bat records occurring in each grid cell and then classified them as either “sampled” (with at least one record of any species) or “un‐sampled” (without any record).

To illustrate the geographic variation in sampling activity, we created a map showing a network of Thiessen polygons based on the centroids of all sampled grid cells. This is a scale‐independent way of illustrating variation in sampling activity where a big polygon indicates regionally low sampling activity, whereas a small polygon indicates the opposite (Schulman, Toivonen, & Ruokolainen, 2007; Vale & Jenkins, 2012). This analysis was done using the “Create Thiessen Polygons” tool in ArcGIS v 10.3.

Then, based on the grid cells representing the sampling effort, we assessed how well the environmental conditions in Mexico were sampled by quantifying the spatial sampling bias with respect to the main ecoregions in the country following the formula developed by Kadmon et al., (2004) (Equation 1). The use of ecoregions allowed us to define geographic units that share similar environmental and ecological conditions and are recognized units for global conservation prioritization (INEGI, CONABIO, & INE, 2008).

(1) $${\text{Bias}}_{d}=\frac{n_{d}-p_{d}N}{\sqrt{p_{d}\left(1-p_{d}\right)N}}$$

where *n*_d_ is the number of grid cells with bat records in the ecoregion d, *p*_d_ is the proportion of the grid cells in Mexico which fall in ecoregion d, and *N* is the total number of sampled grid cells (*N* = 2,289). To estimate *p*, a set of *N* grid cells (with *N* equal to the number of actual sampled grid cells) was distributed at random in Mexico, and then *p* was taken to be the fraction of the random grid cells within a particular ecoregion. To estimate the number of random grid cells, we generated 100 sets of random grid cells of the same number as the sampled grid cells and calculated the mean across the 100 runs per ecoregion. The number of sampled grid cells by ecoregion was then compared with number of random grid cells using a chi‐squared test to assess whether sampled grid cells fell into significantly (*α* = .05) different proportions of ecoregions than expected by chance.

We analyzed relationships between the availability of records and seven explanatory variables related to species richness, accessibility, and security level at a resolution of ~10 km^2^ grid cells. Three explanatory variables represent species richness: (a) bat species richness, that is, the number of all species that are expected to occur in each grid cell, estimated by overlaying the IUCN range maps of all the bat species distributed in Mexico (IUCN, 2015) and counting how many coincide in each cell, (b) elevational heterogeneity was estimated as the difference in elevation within each grid cell based on a digital elevation model at 60 m resolution (INEGI, 2017), and (c) the coverage of federal protected areas estimated as the percentage of the protected areas polygons within each grid cell (CONANP, 2016). Other three variables represent the accessibility of each cell: (a) road density estimated as the sum within each grid cell of roads length in km (INEGI, 2015), (b) percentage of urban areas estimated as the percentage of the city polygons within each grid cell (INEGI, 2014), and (c) human population density by municipality estimated by assigning the value or average of values given by the map polygons that coincide in each cell (CONABIO, 2010). The remaining variable measures the security level of each grid cell, using reports of the Centro Nacional de Información, which aggregates information on average rate of homicides between 1996 and 2000 per state (SEGOB, 2016). All variables had a correlation less than 0.6 or −0.6.

To address spatial autocorrelation in residuals, we used conditional autoregressive (CAR) models in this analysis. Considering the zero‐inflated nature of the data (i.e., 23,544 out of 25,833 grid cells have no records), we used two response variables in separate regression analyses: (a) the presence of records in each grid cell (*n* = 25,833) and (b) the number of records in each of the grid cell with records (*n* = 2,289). In both models, the explanatory variables were the seven variables described above. All explanatory variables were standardized (mean = 0 and *SD* = 1) before model fitting. We fitted the models to the data with the Markov chain Monte Carlo (MCMC) method in OpenBUGS 3.2.3 (Spiegelhalter, Thomas, Best, & Lunn, 2014) and the R2OpenBUGS package (Gelman et al., 2017) in R (R Development Core Team, 2013). Prior distributions of parameters were set as noninformatively as possible, so as to produce estimates similar to those generated by a maximum likelihood method. We used normal distributions with mean of 0 and variance of 1,000 for coefficients of explanatory variables, an improper uniform distribution (i.e., a uniform distribution on an infinite interval) for the intercept as recommended by Thomas, Best, Lunn, Arnold, and Spiegelhalter (2004), and a Gamma distribution with mean of 1 and variance of 1,000 for the inverses of variance in an intrinsic Gaussian CAR distribution. Each MCMC algorithm was run with three chains with different initial values for 100,000 iterations with the first 50,000 discarded as burn‐in and the remainder thinned to one in every 10 iterations to save storage space. Model convergence was checked with R‐hat values (Gelman, Carlin, Stern, & Rubin, 2003).

### 2.4 Priority survey areas

We conducted univariate analyses for the relationship between the presence of records in each grid cell (*n* = 25,833) and each of the four threat variables (i.e., predicted changes by 2050s in mean temperature and precipitation, forest and farmland cover percentage per grid cell) using CAR models to address spatial autocorrelation in model residuals, which is highly expected in this type of data. To generate the risk variables, we obtained mean values of annual precipitation and annual temperature at a 5 arc minutes resolution (Hijmans, Cameron, Parra, Jones, & Jarvis, 2005). For future conditions, we selected the CCSM4 General Circulation Model and an extreme greenhouse gas concentration trajectory (Representative Concentration Pathways—RCP) for 2050s with CO^2^ predicted to reach around 2,000 ppmv (RCP‐8.5) (IPCC, 2013). Current and future land use maps were obtained from van Eupen et al., (2014). For future land conditions, we selected two extreme socioeconomic contexts: (a) a sustainable scenario where the use of resources and fossil fuels and deforestation is reduced; and (b) a “business‐as‐usual” scenario where no efforts to reduced land degradation and the use of fossil fuels are taken. We used four land use classes (forest, shrubland, grassland, and cropland) at 5 arc minute resolution and estimated their proportions per each grid cell.

## 3 RESULTS

### 3.1 Sampling biases

**Figure 3:**
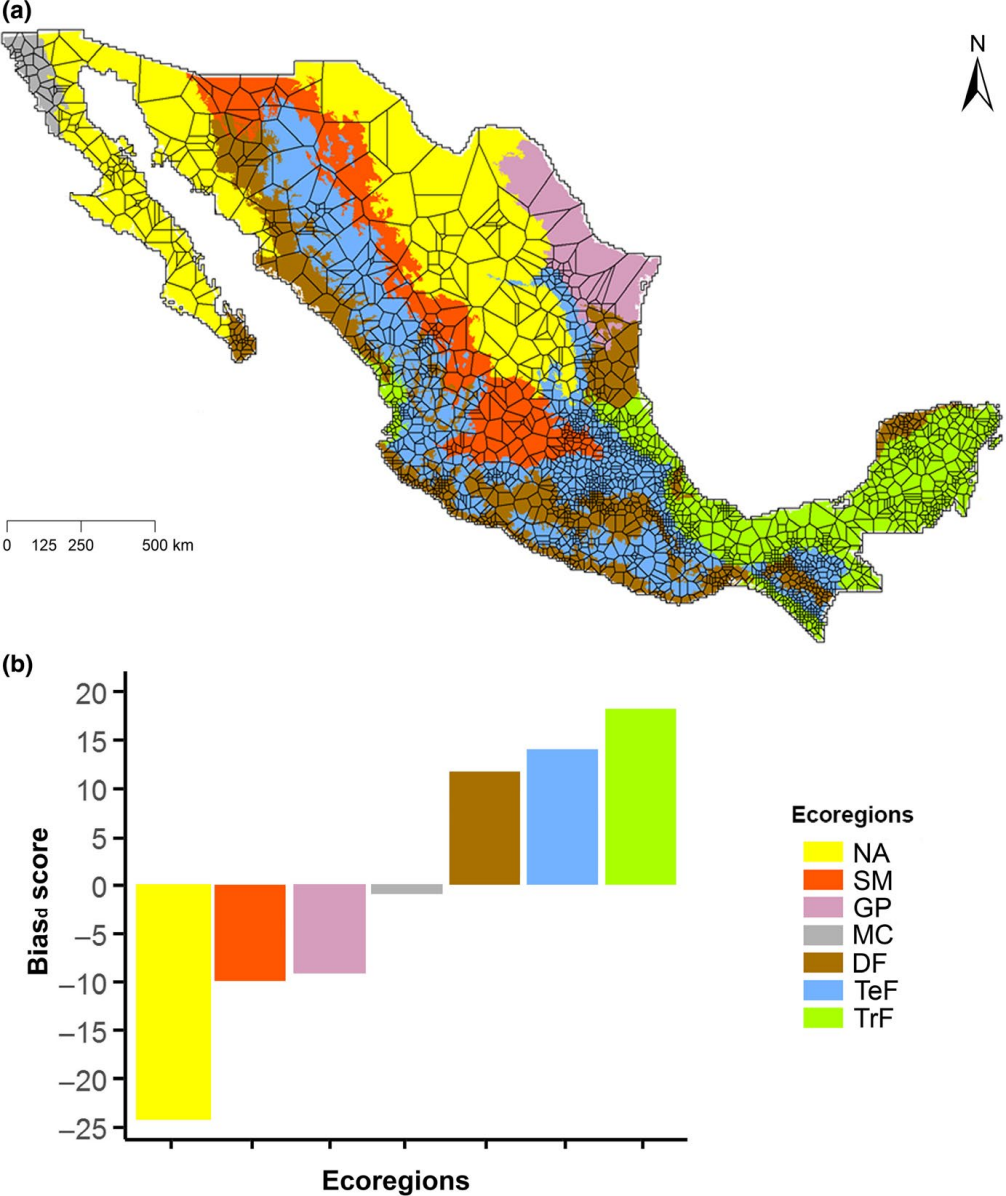
Spatial and environmental representation of sampling bias. (a) Sampling activity as the area of Thiessen polygons centered on the centroid of each sampled grid cell. Each polygon shows the area closest to the sampled centroid that is situated in the center of the polygon. The smaller the polygons, the higher the density of sampled grid cells. Colors represent the ecoregions at level I defined by INEGI, CONABIO, & INE, (2008). (b) Environmental bias (Bias_d_) in sampled grid cells for Mexico across ecoregions. Bias values go from positive values which indicate the over‐sampling, to negative values which indicate environmental under‐sampling. DF, Deciduous Forest; GP, Great Plains; MC, Mediterranean California; NA, North American Deserts; SM, Semiarid Meridional Elevations; TeF, Temperate Forest; TrF, Tropical Forest

Thiessen polygons network showed that the knowledge of the bat fauna in the North American deserts and the great plains is based on collections from a few widely spread sampled grid cells (Figure 3a). The area of the largest polygon is more than 48,000 km^2^, indicating that knowledge of bats in this area is based upon a single sampled grid cell. There were 31 of these large polygons (≥10,000 km^2^) covering more than 59 million km^2^, 16 of which were located in the North American desert ecosystem, six in the great plains, four in the north of the western mountain range and five in the north of the semiarid meridional elevations. Supporting this result, the proportion of sampled grid cells in different ecoregions was significantly different from that expected by chance (*x* ^2^ = 645.64, *df* = 6, *p* < .001). The calculation of Bias_d_ revealed that three ecoregions, North American deserts, semiarid meridional elevations and great plains, have particularly been under‐sampled while three ecoregions, tropical forest, temperate forest, and deciduous forest, have been over‐sampled (Figure 3b).

The number of reported species per sampled grid cell was very low with 68% of the sampled grid cells having ≤5 species reported. The percentage of all grid cells within a species' range that were sampled ranged between 0.1% and 27%, with 50 (37%) species having ≤20 grid cells sampled. There was a significant effect of range size, foraging space, and their interaction on the number of sampled grid cells (Table 1, Model 1). Post hoc comparison showed that number of sampled grid cells for narrow‐space foragers was, when controlled for their range size, significantly higher than those for edge‐ and open‐space foragers, while there was no significant difference between edge‐ and open‐space foragers (Table 1, Model 2). The estimated exponent *b* in the relationship *y* = *a x^b^* (*y* = the number of grid cells sampled, *x* = range size) was significantly smaller than 1 for open‐space foragers and for edge‐space foragers, while it was not significantly different from 1 for narrow‐space foragers (Table 1, Model 3). This indicates that the proportions of grid cells sampled were higher in narrow‐ranging species for open‐and edge‐space foragers.

**Table 1:** Results of the linear model comparing the effects of range size (log‐transformed and centered) and foraging space (edge‐space forager as a reference category) and their interaction on the number of grid cells sampled (log‐transformed) in each species (Model 1)

	Model 1			Model 2				Model 3	
	*df*	*F*	*p*	Estimate	*SE*	*t* value	*p*_adj_	*b*	95% CI
Range size	1,128	177.80	<.001						
Foraging space	2,128	12.78	<.001						
Interaction	2,128	3.28	<.04						
N‐E				0.63	0.20	3.22	0.004		
O‐N				−1.03	0.23	4.49	0.001		
O‐E				−0.40	0.25	−1.63	0.23		
O								0.53	0.26–0.79
E								0.71	0.55–0.86
N								0.94	0.74–1.15

Note

Model 2 shows the results of the Tukey HSD post hoc test for a multiple comparison between the foraging groups (O = open‐space foragers, E = edge‐space foragers, N = narrow‐space foragers). Model 3 summarizes the results of Model 2 with an additional estimate of the slope of log‐transformed range size and its 95% confidence interval for each foraging space group. This slope can be interpreted as the exponent *b* of a power–law relationship: *y* = *a x^b^*, where *y* represents the number of grid cells sampled and *x* range size (i.e., the number of grid cells in which the species is expected to occur).

### 3.2 Predictors of spatial bias

**Figure 4:**
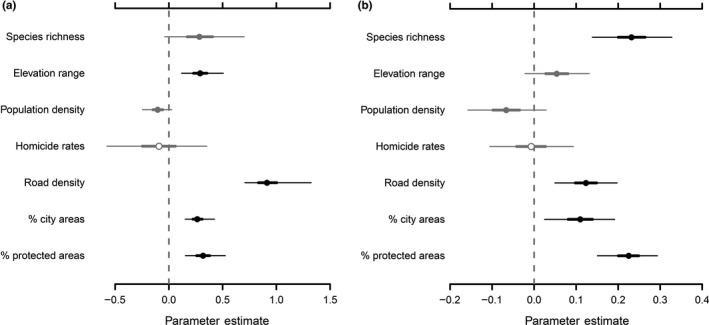
Effects of hypothesized explanatory variables on (a) the presence of records in each grid cell (*n* = 25,833); and (b) the number of records in each of the grid cell with records (*n* = 2,289). Estimated coefficients (posterior medians) with 95% and 50% (thick lines) credible intervals are shown (those not overlapping with zero shown with filled circles in black)

Results of CAR models indicate that both the attractiveness and accessibility of grid cells are important predictors of data availability. The presence of records in each grid cell was associated particularly with high topographic heterogeneity, road density, percentages of city area, and protected areas (Figure 4a). Similarly, among grid cells with records, there were more records in grid cells with higher species richness, road density, and percentages of city areas and protected areas (Figure 4b).

### 3.3 Priority survey areas

**Figure 5:**
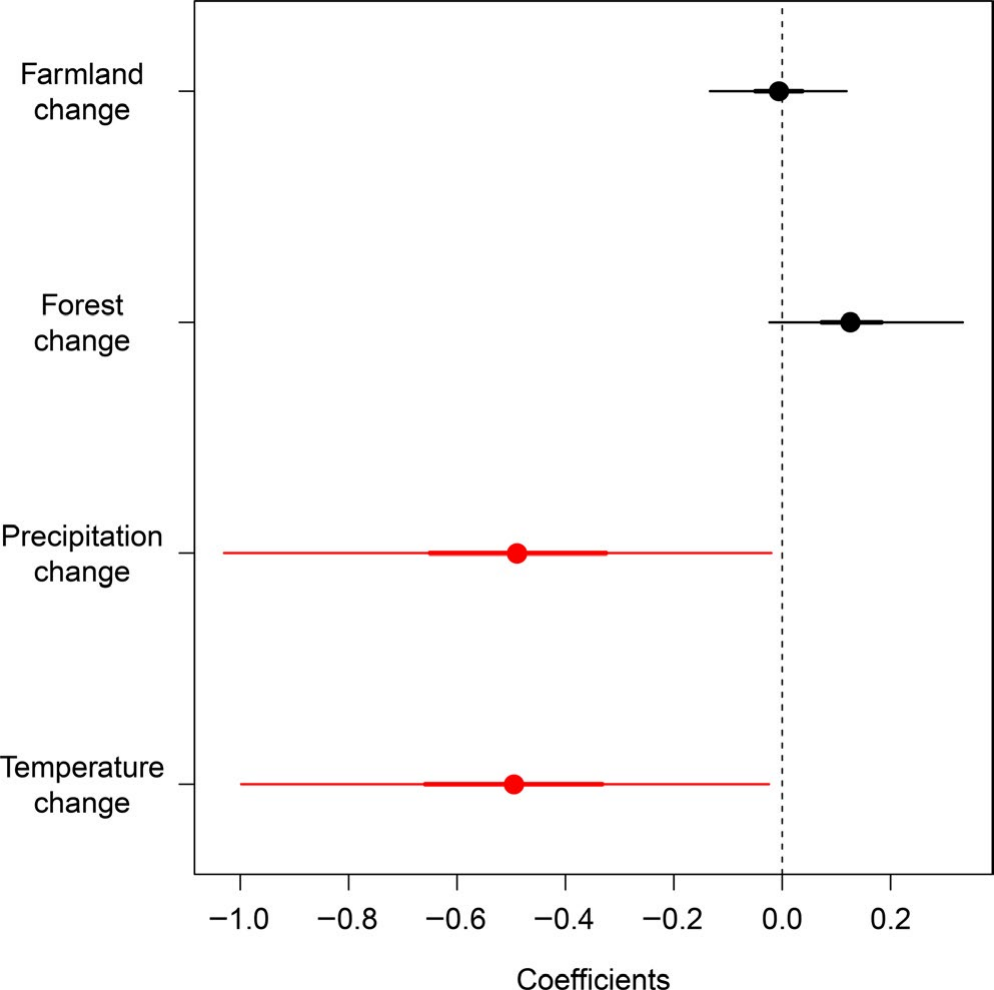
Estimated coefficients in univariate analyses testing the relationship between the presence of records and each of the four threat variables: predicted changes in temperature, precipitation, forest cover and farmland area. Posterior medians with 95% and 50% (thick lines) credible intervals are shown. Coefficients with 95% credible intervals not overlapping with zero are shown in red

The presence of records was negatively associated with changes in temperature and precipitation (Figure 5), indicating that records are particularly scarce in areas with predicted increases in temperature and precipitation. On the other hand, the presence of records was associated with neither forest change nor farmland change (Figure 5). Nevertheless this means that areas with predicted deforestation and farmland area expansion are not necessarily surveyed well compared to other areas. Priority areas for further surveys which are currently under‐sampled and at higher risk of environmental change include the North American Deserts, Semiarid Meridional Elevations and the Great Plains.

## 4 DISCUSSION

Although the dataset collated here represents the most comprehensive set of occurrence records for Mexican bats, it shows that Mexico is poorly sampled with 90% of the territory with no bat occurrence records and with a sampling effort unevenly distributed across the country. The substantial lack of biodiversity data coverage in megadiverse regions has been pointed out in previous studies (Amano & Sutherland, 2013; Bernard, Aguiar, & Machado, 2011; Collen, Ram, Zamin, & McRae, 2008; Llorente‐Bousquets, Ocegueda, Contreras, Chiang, & Papavero, 2008) but this is the first time such detailed analysis has been done for Mexican bats. The sampling gaps found in Mexico are a matter of concern as they could substantially influence the way ecological and biodiversity value of different parts of the country are perceived. If patterns and changes in biodiversity cannot be measured accurately, threats to species and ecosystems cannot be adequately addressed and consequently conservation efforts might be misdirected (Collen et al., 2008).

### 4.1 Data reliability

Spatial sampling bias over different ecoregions was evident in the existing bat occurrence records in Mexico. Sampling appears to have focused disproportionately on the tropical regions, while the bat fauna of the drylands are represented by only a few scattered sampled grid cells. Hence, the completeness of the knowledge of the Nearctic and the Neotropical bat faunas is unlikely to be equal. This pattern of over and under‐sampling of some ecoregions has been previously identified in other temperate and arid regions (Bernard et al., 2011; Martin, Blossey, & Ellis, 2012; Meyer, Kreft, Guralnick, & Jetz, 2015). Bats are crucial components of these under‐sampled environments as they provide key ecosystem services and have high mammalian richness and abundance (Carpenter, 1969; Jones et al., 2009). Thus, the uneven coverage in the knowledge of the bat faunas may have important consequences in safeguarding the natural capital of these regions and the provision of services to the human well‐being (e.g., biological insect pest suppression).

Mexico has the highest percentage of bat occurrence records from Latin America (37%) in the GBIF portal (Noguera‐Urbano & Escalante, 2014). Yet, more than 50% of the sampled grid cells have five or less species recorded and none of the species had more than 30% of its range sampled. Despite the large efforts by Mexican institutions to increase the availability of electronic verified records (Soberón & Peterson, 2004), data quantities for a large number of species are still insufficient. Although the efforts from different sources and researchers have allowed us to achieve some understanding of the distribution of richness and species for Mexican bats, this picture might not be a fair representation of the true distributions (Sastre & Lobo, 2009). When species richness is predicted using biased data, the predicted values tend to be higher in the most surveyed localities compared to those under‐sampled. Hence, biased sampling effort can also bias estimates of species richness (Petřík et al., 2010; Sánchez‐Fernández, Lobo, Abellán, Ribera, & Millán, 2008).

The analysis presented here shows that the representation of bat species in the occurrence records was influenced not only by species' range size but also by their foraging behavior. The effect of foraging style on the amount of sampled grid cells could be explained by two main reasons: (a) the over‐sampled tropical ecosystems might have been dominated by narrow‐space foragers (Fenton et al., 1992); or (b) narrow‐space foragers are the best detected group by capture methods (mist nets) which are preferably used in the Neotropics (Barnett et al., 2006). Mist nets undoubtedly favor the detection of some narrow‐space foragers, such as phyllostomid species and under‐sample open‐ and edge‐space foragers (e.g., molossids and vespertilionids) (MacSwiney et al., 2008). However, recent technological innovations have led to the increased use of new survey methods (e.g., ultrasonic detectors) (Jones et al., 2013) that could change the overall representativeness of species sampled over time. However, changes in species detectability caused by changes in survey methods may lead to erroneous assumptions about species distributional changes. It is important to further explore effects of survey methods on species data representativeness in order to disentangle changes attributable to sampling rather than environmental or ecological drivers (see da Rocha, Ferrari, Feijó, & Gouveia, 2015), and to apply correcting methods when dealing with biased data. For example, the information contained in occurrence records can be improved (without losing data) with some recently developed statistical techniques like random walk priors in Bayesian occupancy models (Outhwaite et al., 2018), and specific target‐group background selection methods when building SDMs (Phillips et al., 2009).

### 4.2 Drivers of bias

We found that variables that favor high species richness within an area (i.e., protected areas and topographic heterogeneity) had a positive effect on the availability of bat occurrence data. This result is consistent with previous studies where sampling effort is biased toward regions considered as conservation priorities (Parnell et al., 2003; Reddy & Dávalos, 2003; Yang, Ma, & Kreft, 2014). Plausible explanations include that protected areas are more secured, which is a considerable barrier for researchers in countries under conflict (Amano & Sutherland, 2013). Also, protected areas are more likely to host pristine habitats which might increase the chances to find endemic, rare, and a higher number of species. Biases toward areas with perceived high species richness can be particularly problematic for conservation purposes because it can give the wrong impression of certain areas to be species rich, which can lead to misleading prioritization for conservation planning (Boakes, Fuller, McGowan, & Mace, 2016).

The concentration of bat occurrence data in highly heterogeneous habitats like mountains can be explained by three main factors. First, the most important institutions for biodiversity and taxonomic research are located in Mexico city and toward the center and south of the country (Llorente‐Bousquets, Michán, et al., 2008; Llorente‐Bousquets, Ocegueda, et al., 2008). These institutes are located close to areas of high mammal's biodiversity and endemism like the states of Oaxaca, Chiapas, and Veracruz (Ceballos & Oliva, 2005). Second, these biodiverse states host important and complex mountainous areas in Mexico like the Sierra Madre de Chiapas and Sierra Madre del Sur, and unique vegetation types like the cloud forest. Third, the east and west mountain ranges in the country are considered between having medium to high mammal's diversity (Ceballos & Oliva, 2005). We can thus conclude that the highly biodiverse, pristine, and heterogeneous mountainous areas of Mexico have attracted the attention of trained taxonomist over the years (see references within Ceballos & Oliva, 2005).

Location's accessibility is also an important predictor of data availability for both presence and abundance of records, meaning that more accessible places tend to be more frequently sampled and with more data. These biases in occurrence data could lead to erroneous ecological patterns. For example, previous studies have found that off‐road avian community composition significantly differs from samples taken around roads (Wellicome, Kardynal, Franken, & Gillies, 2014). Similar patterns might hold for bats as different species and trophic groups have different tolerance to disturbance and human‐made environments (Estrada‐Villegas, Meyer, & Kalko, 2010; Gonçalves, Fischer, & Dirzo, 2017; Jung & Kalko, 2011). Sampling bias toward densely populated areas can cause erroneous conclusions about associations between species and people, and it may underestimate the impact of human activities on biodiversity (Barbosa, Pautasso, & Figueiredo, 2013; Ficetola et al., 2014). Mexico leads the list of countries with the highest deforestation rates (Mas et al., 2004) and with the highest human densities (World Bank, 2017). The biases found here toward anthropogenic elements give caution in making ecological and biogeographic inferences on bat distributions as it might have serious consequences for conservation and management plans. It is important to study data biases involving human‐made environments to disentangle sampling artifacts from environmental patterns and associations. Security aspects may also influence field sampling but we did not find evidence that insecure conditions limit the number of records available in Mexico. This result might be scale dependent as we used a security metric given at state level. A more detailed analysis on specific dangerous areas might give a different result as it is known that scientist tend to avoid sampling conflict areas (Amano & Sutherland, 2013; Boakes et al., 2016).

### 4.3 Prioritization

This study identified the areas that need to be better explored and characterized in terms of their bat fauna. Our study emphasizes the need to improve bat data, and very likely from other taxa, in areas with specific environmental (drylands), geographic (northern Mexico and outside protected areas), and socio‐economic characteristics (e.g., remote away from cities and roads), and for specific bat groups (e.g., open‐space foragers) to improve the quality and representativeness of distributional data and consequently species knowledge to take well‐informed conservation decisions. The sampling biases identified here lead to an evident Wallacean shortfall, that is, poorly understood and incomplete knowledge on species distributions. It is particularly worrying that the lack of records is found in areas projected to have extreme weather events by 2050s and that are likely to suffer high rates of land use change. This lack of information is likely to limit (a) our understanding on how biodiversity is and will be affected by environmental changes and (b) subsequently our capacity to develop sound conservation strategies.

Ideally, species should be sampled homogenously across space and time and a good example of long term and long scale biodiversity surveys can be seen at the Butterfly Monitoring Scheme which started in the UK in the mid‐1970s, and since then, it has been extended to more than ten European countries. This monitoring program is based on standardized sampling protocols designed to collect data for both common and rare species, covering a wide range of habitats, and it is constantly updated and adjusted to detect and reduce data gaps by prioritizing survey efforts (Van Swaay, Nowicki, Settele, & Strien, 2008). This type of surveys must be implemented in countries highlighted as data deficient and of high conservation priorities like Mexico (e.g., Brazil, Bernard et al., 2011).

The next step is to fill in the information gaps identified here. Mexico is leader in data mobilization through the CONABIO (Soberón & Peterson, 2004); thus, this data biases might be a consequence of the lack of sampling effort, rather than data sharing. A crucial action is to prioritize data collection to minimize efforts and money. Citizen science programs have proven to be effective to increase the representativeness of some taxa on digital occurrence databases (Amano, Lamming, & Sutherland, 2016), as they can be done over large regions at a low cost. However, citizen science data collection may continue promoting certain biases like accessibility and security. Overcoming these biases will be particularly challenging in megadiverse regions as the most data deficient regions are less accessible and secure, and require a great investment of time and money to reach (Bernard et al., 2011).

Indirect observation through acoustic surveys is particularly useful for less detectable groups like bats and they have been successfully implemented to improve the coverage of bat's distributional data (Jones et al., 2013). In any case, further sampling must be encouraged toward areas and species that require immediate attention. In other to close the information gap across species and regions, new data collection programs should focus on the regions and species groups with data deficiencies identified here. Survey design should be stratified so that sampling must be systematic and carried out by trained biologists or surveyors.

## CONFLICT OF INTEREST

None declared.

## AUTHOR CONTRIBUTIONS

V.Z.G. and K.E.J. conceived and designed the study, V.Z.G. collected the data, V.Z.G. and T.A. analyzed the data, V.Z.G led the writing with contributions from all authors.

## Data Availability

Data from the variables used for the analysis have been deposited at Dryad: https://doi.org/10.5061/dryad.qrfj6q5b3
